# Long run height and education implications of early life growth faltering: a synthetic panel analysis of 425 birth cohorts in 21 low- and middle-income countries

**DOI:** 10.1186/s12889-019-7203-5

**Published:** 2019-07-04

**Authors:** Mahesh Karra, Günther Fink

**Affiliations:** 1 Frederick S. Pardee School of Global Studies, Boston University, 152 Bay State Road, Room G04C, Boston, MA 02215 USA; 2 Swiss Tropical and Public Health Institute & University of Basel, Socinstrasse 57, 4051 Basel, Switzerland

**Keywords:** Adult height, Child stunting, Demographic and health surveys, Educational attainment, Height-for-age, Low- and middle-income countries

## Abstract

**Background:**

We estimated the associations between exposure to early life growth faltering at the population level and adult height and education outcomes in a sample of 21 low- and middle-income countries.

**Methods:**

We conducted a synthetic panel analysis of 425 birth cohorts across 126 regions in 21 LMICs surveyed in the Demographic and Health Surveys (DHS) both as children and as adults. Data from historic (1987–1993) DHS survey rounds were used to compute average height-for-age z-scores at the province-birth-year level. Cohort measures of early life growth were then linked to adult height and educational attainment measures collected on individuals from the same cohorts in the 2006-2014 DHS survey rounds. The primary exposure of interest was population-level early life growth (region-birth year average HAZ) and growth faltering (region-birth year stunting prevalence). Multivariable linear regression models were used to estimate the associations between adult outcomes and population-level measures of early life linear growth.

**Results:**

The average cohort height-for-age z-score (HAZ) in childhood was − 1.53 [range: − 2.73, − 0.348]. In fully adjusted models, each unit increase in cohort childhood HAZ was associated with a 2.0 cm [95% CI: 1.09–2.9] increase in adult height, with larger associations for men than for women. Evidence for the association between early childhood height and adult educational attainment was found to be inconclusive (0.269, 95% CI: [− 0.68–1.22]).

**Conclusions:**

While early childhood linear growth at the cohort level appears to be highly predictive of adult height, the empirical association between early life growth and adult educational attainment seems weak and heterogeneous across countries.

**Registration:**

This study was registered on May 10, 2017 at the ISRCTN Registry (http://www.isrctn.com), registration number ISRCTN82438662.

**Electronic supplementary material:**

The online version of this article (10.1186/s12889-019-7203-5) contains supplementary material, which is available to authorized users.

## Background

In 2010, more than 167 million children (25.6%) under the age of five in low- and middle-income countries (LMICs) were estimated to be stunted [1]. Progress towards averting child stunting has been slow, particularly in South Asia and in parts of sub-Saharan Africa where most early life growth faltering occurs today [1, 2]. While growth faltering among children under age five has increasingly been used as a proxy for children’s overall early life development as well as for their long term potential [3, 4], evidence on the links from early life physical growth to cognition or educational outcomes remains mixed. A recent review of long-term cohort studies in LMICs found positive associations between height-for-age z-scores (HAZ) at age 2 and educational attainment in four LMICs but no such association in a South African cohort [5]. Similarly, another recent review of published studies found relative strong cross-sectional associations between cognition and childhood HAZ but found no impact of nutrition-focused interventions on either cognition or schooling outcomes [6]. Evidence from the Dutch Famine of 1944–45 suggests that hunger in the first one or two years may have altered growth trajectories; however, undernutrition after this period may not have altered later-life adult height [7] or long-term risk of mortality [8], although later-life adult morbidity from coronary heart disease was found to be more pronounced in exposed cohorts [9]. Similarly mixed results were also found for historical early life hunger studies from the Nazi Seige of Leningrad [10] and the Biafran War in Nigeria [11]. Finally, a large number of studies examining the Chinese Famine of 1959–1961 have assessed the relationships between early-life exposure to severe undernutrition and adult height and stunting [12–14], mental health [15], educational attainment [13, 16], labor supply [12, 13, 16], and earnings [12]. Estimations from these studies have found a wide range of results of the consequences of the famine, recorded to be the worst in human history [17], on adult outcomes.

Conceptually, the lasting impacts of early life linear growth on adult outcomes seem plausible if children’s cognitive and physical development (epigenetically) are able to adjust to resource-scarce environments [18]. A significant body of work has suggested that that stunting may be largely irreversible after the first 1000 days from conception to a child’s second birthday, leading to an intergenerational cycle of poor growth and development [19, 20]. However, other studies have also shown evidence of “catch-up growth” in adolescence following early-life stunting [21], and even in the absence of interventions [22]. While there is a likely strong correlation between childhood and adulthood height [5, 23, 24], these individual-level correlations may reflect, to a large extent, genetic differences that affect both childhood and adult outcomes and should therefore not be interpreted as evidence for a causal effect of nutrition on development.

From a measurement perspective, the extent to which children are exposed to malnutrition, poverty, poor water and sanitation, and adverse environments in early life can most easily be measured at the cohort level, where population reference tables exist. [25, 26]. Populations with significant exposure to early life infections and undernutrition, especially in sub-Saharan African settings, typically display average HAZ scores close to zero during the exclusive breastfeeding period (typically within the first 6 months postpartum), followed by a rapid decline in childhood HAZ up to age 2, at which point the population-level childhood HAZ seems to stabilize despite the large variation in individual growth trajectories [22, 27, 28].

In this paper, we extract all available anthropometric data collected since 1986 as part of the early rounds of the Demographic and Health Surveys (DHS) program to estimate associations between early life linear growth environments and adult outcomes.

## Methods

This study constructs a synthetic panel of cohorts to assess the long-run association between childhood stunting and adult height and educational attainment. All data used in this study come from the DHS, which are nationally- and sub-nationally representative household surveys that provide information on a wide range of indicators in the areas of population, maternal and child health, and nutrition. More than 300 DHS surveys have been collected in over 90 countries since 1984 [29]. To construct our synthetic panel, we use data from historic DHS survey rounds to compute average height-for-age z-scores at the birth cohort level. Birth cohorts are defined as all children who are born in a given region or province of a country, as defined by the DHS, in a given year (e.g. all children born in 2002 in Barisal region, Bangladesh). We then link our computed cohort measures of early life growth to adult height and educational attainment measures that are collected on individuals from the same cohorts in subsequent DHS survey rounds. Given the focus of this study, we restricted our analysis to countries that surveyed the same populations for at least 15 years and that included both an initial anthropometric assessment at childhood and at least one adult (age 21 or older) assessment. DHS programs generally conduct anthropometric assessments for children under the age of five; however, since population-level growth faltering can only be fully observed around age 2 [27], we exclude children under age 2 from our cohort-level growth faltering analysis. In addition, we pool the HAZ and stunting estimates of all children aged 2–4 who were from the same region and who were surveyed in a given round in order to gain precision in our growth faltering assessment. For example, for a survey that was conducted in 1993, we pool together children aged 2–4 (born between 1989 and 1991) from the same region in our construction of cohort-level HAZ and stunting rates. We also considered a finer-grained model that calculated average HAZ measures that were computed for each birth cohort; however, the resulting sample sizes were often very small and the HAZ estimates for individual birth cohorts were too noisy (with estimated stunting prevalence between 0 and 100%) to allow for meaningful statistical inference due to the small cell sizes at the country-region-individual birth cohort level. Finally, when considering the relatively large proportion of individuals who were still in school at age 18, we focused on the sample of adults aged 21 and older in order to obtain a more complete measure of adult educational attainment as an outcome.

### Statistical methods

We use multivariable linear regressions to estimate the associations between average height-for-age in childhood and adult height as well as to estimate the associations between average height-for-age in childhood and educational attainment in adulthood, the latter of which is measured by years of schooling. In addition, we run multivariate logistical regressions to measure the association between average height-for-age in childhood and stunting in adulthood, which is measured as a binary variable. Our models include both adult-level covariates that are observed in the post-2000 surveys as well as childhood-level covariates that are observed in the 1980 and 1990s. Our adult- and child-level covariates aim to capture key determinants of child growth and development that have been identified in the literature, particularly maternal education, place of residence, socioeconomic status, and exposure to poverty [26]. Adult covariates include age, sex (male/female), place of residence (urban/rural), household size at adulthood, and an estimate for logged income per capita. Childhood covariates include the percentage of children in rural areas, average maternal education in the region, the percentage of children living in households with access to electricity, and the estimated average level of income per capita of their households. We include country-specific intercepts in our model as fixed effects in order to ensure our results are not affected by country-level trends, and we correct for cohort-level correlations in the exposure variables by clustering our standard errors at the country-region-survey level using Huber’s cluster-robust variance estimator [30]. Additional file 1: Table A1 in the Additional file provides additional description of the variables that are used in our analysis. All analyses were performed using Stata, version 13 [22].

## Results

A total of 454,280 child records from 3885 cohorts were extracted to compute early life growth measures, and a total of 126,584 adult records were successfully linked to cohort-level child anthropometric measures. The oldest cohorts were born in 1985, and the youngest cohorts were born in 1993. After dropping 22,225 observations due to missing income data, our final analytic sample is comprised of 104,359 adult records representing 425 birth cohorts surveyed between 2006 and 2014 in 21 LMICs. Adult height was available for a total subsample of 33,998 adults. Additional file 1: Figure A1 further illustrates the process by which the analytic samples were constructed from the original DHS data. Additional file 1: Table A2 presents a list of the countries and birth cohorts that are included in the final analytic sample, and Additional file 1: Figure A2 in the presents a map of the countries that are included in the final sample.

**Fig. 1:**
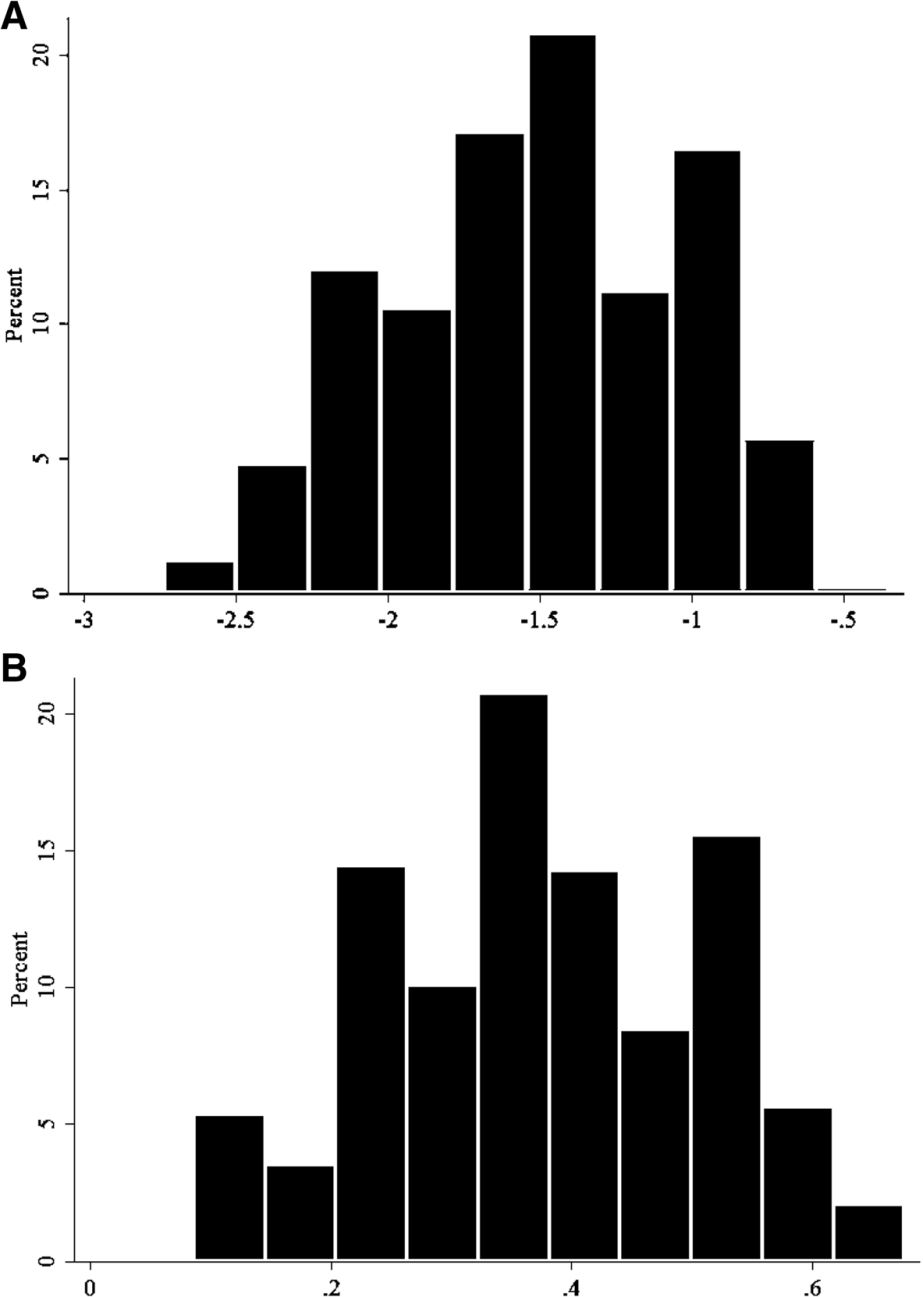
Average Height and Stunting Prevalence in Childhood, Cohort Averages. Panel **a**: Cohort Average Under-5 HAZ. Panel **b**: Country-Region-Cohort Average Stunting Prevalence Distribution. The figure shows the average cohort-level HAZ as well the proportion of children under 5 who are stunted (HAZ < − 2) across 425 cohorts and in 126 sub-national regions across 27 DHS surveys that were conducted between 2006 and 2014 in 21 LMICs

Figure 1 shows the empirical distribution of average childhood height-for-ages for our full analytic sample of 425 birth cohorts. The average cohort HAZ in childhood was − 1.53 (bottom panel), and average cohort stunting rate in childhood was 37.9%.

Table 1 presents descriptive statistics for the analytic sample. On average, 40% of children in each region lived in urban areas in childhood. The average level of maternal education was 4.5 years at the time of childhood, and the average per capita income in constant purchasing-power-parity adjusted US dollars was I$2.49 per day, implying that the majority of the children lived with incomes below the poverty line in childhood.

**Table 1:** Descriptive Statistics

	*N*	*N*_*1*_	Mean	SD
Adult Characteristics				
Educational attainment (years)	104,359		7.584	5.111
Adult height (cm)	33,998		158.942	8.008
Adult height, men (cm)	3584		171.016	7.341
Adult height, women (cm)	30,414		157.519	6.793
Respondent age (years)	104,359		23.450	1.971
Female (1 = yes)	104,359	56,137	0.538	
Urban (1 = yes)	104,359	51,258	0.491	
Household size	104,359		6.607	4.079
Average log household income per capita	104,359		1.501	1.789
Cohort Childhood Characteristics				
Under-5 childhood HAZ	425		−1.525	0.464
Under-5 childhood stunting (1 = stunted)	425	162	0.379	
Pct. living in urban areas	425	172	0.403	
Avg. maternal education	425		4.499	2.328
Pct. living in household with electricity	425	140	0.328	
Average log household income per capita	425		0.911	0.720

Notes: For binary variables, N_1_ is the number of observations that responded “yes” to the variable, and the mean of the binary variable is the proportion of observations reporting “yes”, which is calculated by dividing N_1_ by the total number of observations N

The average age among adults in our sample was 23.5 years (Table 1, top panel), and a slightly larger proportion of adults in the full analytic sample were women (53.8%). With this said, a much larger proportion of adults for whom we have height data are women (*N* = 30,414, or 89.4% of the analytic sample for adult height) compared to men (*N* = 3584, or 10.6% of the analytic sample for adult height). The average household size in adulthood was 6.6, and average income per capita in adulthood in constant purchasing-power-parity adjusted US dollars was I$4.49 per day. The average adult height in the sample was 158.9 cm, and average educational attainment among adults was 7.6 years.

Table 2 presents results from our multivariable regression for height. When we allow for country-specific intercepts and control for additional childhood and adulthood confounders, we estimate the association between childhood HAZ and adult height to be 2.01 cm [95% CI: 1.09 cm – 2.92 cm] for each additional unit increase in childhood HAZ (Table 2, column 1). When running a stratified analysis by sex (Table 2, columns 2 and 3), we find larger associations for men, with an estimated increase of 2.92 cm [95% CI: 1.42 cm – 4.42 cm] in adult male height for each additional unit increase in childhood HAZ, and an estimated 1.88 cm [95% CI: 0.87 cm – 2.89 cm] increase in adult female height for each additional unit increase in childhood HAZ- this corresponds to a 0.28 SD increase in adult height for women, and to a 0.4 SD increase in male adult height.

**Table 2:** Multivariable Regressions for the Association between Cohort Average Under-5 HAZ and Adult Height

	[1]	[2]	[3]
VARIABLES	Fully Adjusted Pooled Sample	Fully Adjusted WOMEN	Fully Adjusted MEN
Under-5 HAZ	2.005***	1.878***	2.921***
	(1.086–2.924)	(0.870–2.887)	(1.423–4.419)
Controls			
*Child Cohort Characteristics*			
Pct. urban at childhood	−0.581	− 0.754	0.400
	(−1.930–0.767)	(− 2.245–0.736)	(−1.787–2.587)
Pct. electricity at childhood	0.688*	0.575	1.165**
	(− 0.0299–1.406)	(− 0.495–1.645)	(0.254–2.075)
Avg. mat. Educ. at childhood	0.0220	0.0820	−0.496***
	(−0.288–0.332)	(−0.256–0.420)	(−0.783 - -0.209)
Log income at childhood	−0.577***	−0.523**	− 0.563
	(− 1.008 - -0.146)	(− 0.991 - -0.0545)	(−1.481–0.354)
*Adult Respondent Characteristics*			
Female (1 = yes)	−12.06***		
	(−12.65 - -11.47)		
Urban (1 = yes)	1.168***	1.185***	0.930***
	(0.950–1.385)	(0.962–1.409)	(0.265–1.594)
Observations	33,998	30,414	3584
R-squared	0.385	0.167	0.125

*** *p* < 0.01, ** *p* < 0.05, * *p* < 0.1

Notes: The outcome variable in all regressions is adult height in cm. All models are estimated using ordinary least squares, with 95% confidence intervals presented in parentheses. Standard errors are clustered at the country-region-survey year level. Column 1 presents results for the full sample, while columns 2 and 3 present results for the subsample of women and men, respectively

Table 3 shows our multivariable results for adult educational attainment. When we control for country of residence, as well as childhood and adult characteristics (Table 3, Column 1), we estimate a statistically insignificant coefficient of 0.27 years of schooling [95% CI: − 0.68 yrs. – 1.22 yrs] per unit increase of average childhood HAZ. The estimated effect size is small, corresponding to approximately 0.05 SD in educational attainment. Interestingly, this association becomes inverted when we control for adult height (Table 3, Column 2). Our adult height variable in this model displays strong positive associations with educational outcomes; on average, we estimate that an additional inch (2.5 cm) of adult height is associated with about 0.2 additional years of schooling attained. In terms of the childhood covariates that are included, educational attainment in adulthood appears to increase with the average maternal education in childhood and appears to be strongly and positively associated with urban residence in adulthood; in contrast, the relationship between educational attainment in adulthood and urban residence share in childhood displays a strong negative association. Similar to the pooled analysis, we find no significant associations between average childhood HAZ and educational attainment when running a stratified analysis for either men or women (Table 3, Columns 3 and 4). Similar mixed associations were found between childhood stunting prevalence and educational attainment in adulthood once models were adjusted for covariates and country fixed effects (Additional file 1: Table A3).

**Table 3:** Multivariable Regressions for the Association between Cohort Average Under-5 HAZ and Educational Attainment in Adulthood

	[1]	[2]	[3]	[4]
VARIABLES	Fully Adjusted Pooled Sample	Fully Adjusted Pooled Sample + Adult Height	Fully Adjusted Pooled Sample WOMEN	Fully Adjusted Pooled Sample MEN
Under-5 HAZ	0.269	−0.269	0.308	0.315
	(−0.676–1.215)	(−1.663–1.125)	(−0.760–1.376)	(−0.540–1.170)
Controls				
*Child Cohort Characteristics*				
Pct. urban at childhood	−2.874***	−3.646***	− 3.538***	− 2.048***
	(− 3.988 - -1.759)	(−5.339 - -1.954)	(− 4.792 - -2.283)	(− 3.121 - -0.975)
Pct. electricity at childhood	0.761	0.319	0.562	0.857*
	(−0.234–1.755)	(− 0.878–1.517)	(−0.660–1.784)	(−0.0280–1.742)
Avg. mat. Educ. at childhood	0.758***	1.144***	1.015***	0.456***
	(0.456–1.060)	(0.672–1.617)	(0.653–1.376)	(0.225–0.687)
Log income at childhood	0.275	0.0512	0.154	0.404*
	(−0.130–0.681)	(−0.423–0.526)	(−0.296–0.604)	(−0.000473–0.808)
*Adult Respondent Characteristics*				
Female (1 = yes)	−0.953***	0.842***		
	(−1.194 - -0.713)	(0.237–1.447)		
Urban (1 = yes)	2.798***	2.827***	2.973***	2.529***
	(2.479–3.118)	(2.457–3.196)	(2.675–3.271)	(2.168–2.890)
Adult height (cm)		0.0783***		
		(0.0678–0.0887)		
Observations	104,359	33,998	56,137	48,222
R-squared	0.389	0.429	0.443	0.315

*** *p* < 0.01, ** *p* < 0.05, * *p* < 0.1

Notes: The outcome variable in all regressions is attained education in years. All models are estimated using ordinary least squares, with 95% confidence intervals presented in parentheses. Standard errors are clustered at the country-region-survey year level. Column 1 presents results for the full sample, while columns 3 and 4 present results for the subsample of women and men, respectively

Additional file 1: Figure A3 illustrates the general relationship between cohort-level childhood HAZ and adult height as well as adult educational attainment in case of Kenya as an example of a country with larger variations in childhood growth faltering. Similar to the regression results presented in Table 3, we find positive associations between HAZ and educational attainment across regions; this correlation disappears, however, once we condition on maternal education, which appears to be highly predictive of both child HAZ and adult educational outcomes.

We run a series of sensitivity analyses to test the robustness of our findings on adult height and educational attainment. In particular, we: a) re-run our main models with two-way clustering by year of birth and country (Additional file 1: Tables A4 and A5); b) run a sensitivity check by additionally controlling for paternal education (Additional file 1: Tables A6 and A7), which may be correlated with child growth and nutrition [31]; and c) run a sensitivity check where we exclude countries that experienced major shocks over our time period (e.g. Rwanda) that could impact both child growth and long-term adult outcomes (Additional file 1: Tables A8 and A9). All of our sensitivity analyses demonstrate that our main findings are robust to these alternate specifications.

## Discussion

In this study, we construct a synthetic panel using data from 425 birth cohorts across 126 regions in 21 LMICs to evaluate the relationships between early life growth faltering and adult human capital outcomes, namely height and educational attainment. Our results suggest that early life growth at the population level is highly predictive of adult height but is not significantly associated with educational attainment. For height, our results imply that reducing growth faltering to zero in the most malnourished populations studied (average HAZ < − 3) would translate to a long run increase of 8.8 cm in adult male height and a 5.6 cm increase in adult female height. These variations in height seem to be large from a cross-country perspective; a recent study of European populations found a height range of 170 to 179 cm for men and a height range of 160 to 167 cm for women [32]. Given that the average adult in our sample was exposed to substantial growth faltering in childhood, and given that global stunting rates have declined substantially over the past 20 years [33], our findings imply that we may see substantial improvements in adult height in the LMICs that were examined in the coming decades.

In contrast to height, we find no association between early childhood growth and adult educational attainment in our analysis. While our confidence intervals are relatively wide and include the most commonly cited value of a gain in schooling by 0.47 additional years for each unit increase in childhood HAZ [5, 34], the associations between childhood height and adult height seem much more robust and consistent than those observed for educational outcomes. Conceptually, it seems likely that the population-level analysis that we present would yield different results from analyses that focus on individual-level longitudinal data. At the individual level, small height-for-age may not only delay school entry if, for example, minimum height standards for student enrollment are enforced by teachers or other administrators, but may also affect children’s self-confidence, status, and socioemotional development relative to their peers. These individual-level mechanisms are well supported in our data; as Table 3 indicates, taller children do indeed have higher educational attainment, on average. However, the same mechanisms may not apply if, for example, improvements in population-level nutrition that in turn would improve child height were to benefit all children in a similar way such that children’s relative height (and thus also their confidence or socioemotional development) is not affected. If all children were to benefit from roughly the same increases in height, the only pathway through which improvements in childhood HAZ is likely to affect schooling would be through improved cognitive ability. Our results suggest that early life nutritional deficits do not necessarily result in cognitive or educational deficits in later life, which is consistent with the most recent systematic review of early life interventions on child development [35] as well as with recent evidence on the long-run human capital consequences of early life nutrition and stimulation efforts [36].

The study presented has several limitations. First, despite the large dataset that was used for this study and the relatively large number of birth cohorts that were analyzed, the statistical power of the study is somewhat limited due to the population-level aggregation of the child growth data. Most countries in our sample have fewer than 10 regions, which results in somewhat noisy statistical inference once we include county-specific fixed effects and regional controls. A second concern with the study presented is the potential of regional confounders. Even though we control for several socioeconomic factors at the cohort and region level, we cannot rule out residual confounding through other unobservable regional factors, including regional genetic variation, that are correlated with both childhood growth and adult height and educational outcomes. In addition, our data does not allow us to examine catch up growth in height over time for the same children, nor do we have measures of changes in children’s cognitive development over time, which would allow us to make more extensive inferences on the potential lasting impacts of early life deficits in spite of later-life improvements. Finally, our main model rests on the assumption that young adults still live in the region they were born in, which is likely to be true for the majority of the respondents in our sample; however, we cannot completely rule out concerns of migration. Given that migration most typically occurs from less developed rural areas to more developed urban places, it is possible that our estimated associations underestimate the true causal effect of childhood height because some of the rural children who are stunted in childhood would appear in adult urban samples. If it is the case that migration is selective in that the most talented (and tallest) children from the poorest areas migrate to areas with more educational opportunities, then our presented associations would likely be larger than the true causal impact of early life adversity on adult outcomes.

## Conclusions

Overall, the results in this study suggest that early life growth experiences are highly predictive of adult height, but not as much for educational attainment. Further research is needed to better identify the long run health and human capital consequences of early life growth and development.

## Additional file

Additional file 1: Supplemental Materials. (PDF 827 kb)

## Data Availability

All data that are used for this study are available for free download after registering with the DHS Program at http://dhsprogram.com/data/

## Abbreviations

DHS: Demographic and Health Survey
HAZ: Height-for-age
LMIC: Low- and Middle-Income Country
SES: Socioeconomic status
WHO: World Health Organization
