# Supplementary material for: Long run height and education implications of early life growth faltering: a synthetic panel analysis of 425 birth cohorts in 21 low- and middle-income countries
Source: BMC Public Health. 2019 Jul 4;19:876. doi: 10.1186/s12889-019-7203-5 (PMC6611035; doi:10.1186/s12889-019-7203-5)

Supplemental Materials

Table A1: Variable Descriptions

	Variable Description
**Outcome Variables**	
Educational attainment (years)	Years of schooling accumulated by the respondent.
Adult height (cm)	Height of the respondent, in cm.
**Height (Exposure) Variables**	
Under-5 childhood HAZ	Average country-region-cohort level height-for-age z-score (HAZ) in childhood
Under-5 childhood stunting	Country-region-cohort level stunting prevalence (percent of children who are stunted within a given country and region)
**Past Covariates**	
Avg. mat. educ. in childhood	Average maternal education in the region in childhood.
Pct. urban at childhood	Percent of the region that is urban in childhood.
Pct. electricity at childhood	Percent of the region with access to electricity in childhood.
Log income at childhood	Estimated per capita income in the region at childhood.
**Present Covariates**	
Urban (1 = yes)	Binary. Place of residence, either urban (1) or rural (0).
Respondent age (years)	Respondent’s age, in completed years.
Female (1 = yes)	Binary. Whether the respondent is female (1) or male (0)
Household size	Number of household members.
Log income at adulthood	Estimated per capita income at adulthood.

Table A2: Countries and Cohort Years in the Final Sample

Country	Number of Regions	Year	N
Burkina Faso	4	2010	2,229
Cameroon	5	2011	4,692
Dominican Republic	8	2007	1,821
Dominican Republic		2013	4,080
Egypt	5	2008	1,928
Ghana	8	2008	1,319
Ghana	9	2014	2,812
Haiti	3	2012	2,957
Jordan	3	2007	3,485
Jordan		2012	5,401
Kenya	7	2014	8,513
Madagascar	6	2008	963
Malawi	3	2010	5,112
Mali	4	2006	743
Mali		2012	650
Niger	6	2012	3,090
Nigeria	4	2008	3,602
Nigeria		2013	7,516
Pakistan	4	2012	5,729
Peru	16	2009	1,851
Peru		2010	2,246
Peru		2011	2,803
Peru		2012	4,436
Rwanda	5	2010	3,029
Senegal	4	2010	2,847
Senegal		2012	8,485
Senegal		2014	2,618
Tanzania	2	2009	528
Togo	5	2013	921
Uganda	6	2006	413
Uganda		2011	1,339
Zambia	9	2013	6,201
**TOTAL**			**104,359**

Table A3: Multivariable Regressions for the Association between Childhood Stunting and Educational Attainment in Adulthood

VARIABLES	(1) Fully Adjusted Pooled Sample	(2) Fully Adjusted Pooled Sample + Adult Height	(3) Fully Adjusted Pooled Sample WOMEN	(4) Fully Adjusted Pooled Sample MEN
Under-5 stunting	-1.275	0.433	-1.528	-1.308
	(-4.438 - 1.888)	(-4.017 - 4.884)	(-5.110 - 2.055)	(-4.123 - 1.507)
**Controls**				
Child Cohort Characteristics				
Pct. urban at childhood	-2.860***	-3.652***	-3.519***	-2.039***
	(-3.972 - -1.749)	(-5.335 - -1.968)	(-4.768 - -2.270)	(-3.112 - -0.966)
Pct. electricity at childhood	0.755	0.369	0.558	0.846*
	(-0.239 - 1.750)	(-0.788 - 1.526)	(-0.661 - 1.777)	(-0.0413 - 1.732)
Avg. mat. educ. at childhood	0.745***	1.124***	0.997***	0.447***
	(0.447 - 1.042)	(0.660 - 1.589)	(0.641 - 1.353)	(0.218 - 0.675)
Log income at childhood	0.254	0.00789	0.123	0.393*
	(-0.148 - 0.655)	(-0.475 - 0.491)	(-0.329 - 0.575)	(-0.00156 - 0.788)
Adult Respondent Characteristics				
Female (1 = yes)	-0.953***	0.840***		
	(-1.194 - -0.712)	(0.235 - 1.445)		
Urban (1 = yes)	2.798***	2.826***	2.972***	2.529***
	(2.479 - 3.117)	(2.456 - 3.196)	(2.674 - 3.270)	(2.168 - 2.890)
Adult height (cm)		0.0781***		
		(0.0676 - 0.0885)		
**Observations**	**104,359**	**33,998**	**56,137**	**48,222**
**R-squared**	**0.390**	**0.429**	**0.443**	**0.315**

*** $p < 0.01$, ** $p < 0.05$, * $p < 0.1$

Notes: The outcome variable in all regressions is attained education in years. All models are estimated using ordinary least squares, with 95 percent confidence intervals presented in parentheses. Standard errors are clustered at the country-region-survey level. Column 1 presents results for the full sample, while columns 3 and 4 present results for the subsample of women and men, respectively.

Table A4: Multivariable Regressions for the Association between Cohort Average Under-5 HAZ and Adult Height, Two-Way Clustering

VARIABLES	(1)<br>Fully Adjusted<br>Pooled Sample	(2)<br>Fully Adjusted<br>WOMEN	(3)<br>Fully Adjusted<br>MEN
Under-5 HAZ	2.005**	1.878*	2.921*
	(0.153 - 3.857)	(-0.0866 - 3.843)	(-0.580 - 6.423)
**Controls**			
Child Cohort Characteristics			
Pct. urban at childhood	-0.581	-0.754	0.400
	(-2.136 - 0.973)	(-2.449 - 0.941)	(-3.791 - 4.592)
Pct. electricity at childhood	0.688	0.575	1.165
	(-0.210 - 1.586)	(-0.688 - 1.838)	(-0.746 - 3.075)
Avg. mat. educ. at childhood	0.0220	0.0820	-0.496*
	(-0.570 - 0.614)	(-0.541 - 0.705)	(-1.057 - 0.0658)
Log income at childhood	-0.577	-0.523	-0.563
	(-1.351 - 0.197)	(-1.337 - 0.291)	(-1.458 - 0.331)
Adult Respondent Characteristics			
Female (1 = yes)	-12.06***		
	(-13.32 - -10.80)		
Urban (1 = yes)	1.168***	1.185***	0.930*
	(0.748 - 1.588)	(0.739 - 1.632)	(-0.0395 - 1.899)
**Observations**	**33,998**	**30,414**	**3,584**
**R-squared**	**0.385**	**0.167**	**0.125**

*** $p < 0.01$, ** $p < 0.05$, * $p < 0.1$

Notes: The outcome variable in all regressions is adult height in cm. All models are estimated using ordinary least squares, with 95 percent confidence intervals presented in parentheses. Standard errors are clustered using two-way clustering at the country and year of birth levels. Column 1 presents results for the full sample, while columns 2 and 3 present results for the subsample of women and men, respectively.

Table A5: Multivariable Regressions for the Association between Cohort Average Under-5 HAZ and Educational Attainment in Adulthood, Two-Way Clustering

VARIABLES	(1)<br>Fully Adjusted<br>Pooled Sample	(2)<br>Fully Adjusted<br>Pooled Sample +<br>Adult Height	(3)<br>Fully Adjusted<br>Pooled Sample<br>WOMEN	(4)<br>Fully Adjusted<br>Pooled Sample<br>MEN
Under-5 HAZ	0.269	-0.269	0.308	0.315
	(-1.563 - 2.102)	(-2.932 - 2.394)	(-1.766 - 2.383)	(-1.214 - 1.843)
**Controls**				
Child Cohort Characteristics				
Pct. urban at childhood	-2.874**	-3.646**	-3.538**	-2.048**
	(-5.032 - -0.716)	(-7.037 - -0.255)	(-6.071 - -1.004)	(-3.730 - -0.367)
Pct. electricity at childhood	0.761	0.319	0.562	0.857
	(-0.276 - 1.798)	(-1.105 - 1.744)	(-0.550 - 1.675)	(-0.285 - 2.000)
Avg. mat. educ. at childhood	0.758**	1.144**	1.015**	0.456*
	(0.0988 - 1.418)	(0.101 - 2.188)	(0.234 - 1.795)	(-0.0397 - 0.952)
Log income at childhood	0.275	0.0512	0.154	0.404
	(-0.246 - 0.796)	(-0.502 - 0.605)	(-0.344 - 0.652)	(-0.153 - 0.961)
Adult Respondent Characteristics				
Female (1 = yes)	-0.953***	0.842		
	(-1.494 - -0.412)	(-0.981 - 2.665)		
Urban (1 = yes)	2.798***	2.827***	2.973***	2.529***
	(2.175 - 3.421)	(2.151 - 3.502)	(2.384 - 3.562)	(1.841 - 3.217)
Adult height (cm)		0.0783***		
		(0.0585 - 0.0980)		
**Observations**	**104,359**	**33,998**	**56,137**	**48,222**
**R-squared**	**0.389**	**0.429**	**0.443**	**0.315**

*** $p < 0.01$, ** $p < 0.05$, * $p < 0.1$

Notes: The outcome variable in all regressions is attained education in years. All models are estimated using ordinary least squares, with 95 percent confidence intervals presented in parentheses. Standard errors are clustered using two-way clustering at the country and year of birth levels. Column 1 presents results for the full sample, while columns 3 and 4 present results for the subsample of women and men, respectively.

Table A6: Multivariable Regressions for the Association between Cohort Average Under-5 HAZ and Adult Height, Including Paternal Education

VARIABLES	(1)<br>Fully Adjusted<br>Pooled Sample	(2)<br>Fully Adjusted<br>WOMEN	(3)<br>Fully Adjusted<br>MEN
Under-5 HAZ	1.928***	1.726***	2.904***
	(1.037 - 2.819)	(0.755 - 2.697)	(1.427 - 4.380)
**Controls**			
Child Cohort Characteristics			
Pct. urban at childhood	-0.624	-0.870	0.0593
	(-1.935 - 0.688)	(-2.298 - 0.558)	(-2.544 - 2.662)
Pct. electricity at childhood	0.880**	0.890	0.836
	(0.00461 - 1.756)	(-0.298 - 2.078)	(-0.370 - 2.041)
Avg. mat. educ. at childhood	0.113	0.251	-0.576***
	(-0.193 - 0.420)	(-0.0798 - 0.581)	(-0.936 - -0.215)
Avg. pat. educ. at childhood	-0.454	-0.797	0.660
	(-1.442 - 0.535)	(-1.810 - 0.216)	(-0.990 - 2.310)
Log income at childhood	-0.590***	-0.532**	-0.504
	(-1.020 - -0.159)	(-0.998 - -0.0657)	(-1.428 - 0.420)
Adult Respondent Characteristics			
Female (1 = yes)	-12.06***		
	(-12.65 - -11.47)		
Urban (1 = yes)	1.170***	1.190***	0.945***
	(0.952 - 1.389)	(0.965 - 1.414)	(0.262 - 1.627)
**Observations**	**33,998**	**30,414**	**3,584**
**R-squared**	**0.385**	**0.167**	**0.125**

*** $p < 0.01$, ** $p < 0.05$, * $p < 0.1$

Notes: The outcome variable in all regressions is adult height in cm. All models are estimated using ordinary least squares, with 95 percent confidence intervals presented in parentheses. Standard errors are clustered at the country-region-survey year level. Column 1 presents results for the full sample, while columns 2 and 3 present results for the subsample of women and men, respectively.

Table A7: Multivariable Regressions for the Association between Cohort Average Under-5 HAZ and Educational Attainment in Adulthood, Including Paternal Education

VARIABLES	(1)<br>Fully Adjusted<br>Pooled Sample	(2)<br>Fully Adjusted<br>Pooled Sample +<br>Adult Height	(3)<br>Fully Adjusted<br>Pooled Sample<br>WOMEN	(4)<br>Fully Adjusted<br>Pooled Sample<br>MEN
Under-5 HAZ	0.341	-0.0563	0.386	0.373
	(-0.585 - 1.267)	(-1.400 - 1.287)	(-0.658 - 1.429)	(-0.474 - 1.220)
**Controls**				
Child Cohort Characteristics				
Pct. urban at childhood	-2.843***	-3.500***	-3.503***	-2.025***
	(-3.964 - -1.723)	(-5.142 - -1.858)	(-4.778 - -2.228)	(-3.096 - -0.953)
Pct. electricity at childhood	0.360	-0.276	0.128	0.532
	(-0.799 - 1.518)	(-1.726 - 1.173)	(-1.303 - 1.559)	(-0.440 - 1.505)
Avg. mat. educ. at childhood	0.621***	0.886***	0.864***	0.347***
	(0.317 - 0.926)	(0.417 - 1.355)	(0.503 - 1.225)	(0.0978 - 0.595)
Avg. pat. educ. at childhood	0.734	1.297*	0.796	0.594*
	(-0.143 - 1.611)	(-0.193 - 2.786)	(-0.298 - 1.890)	(-0.0892 - 1.277)
Log income at childhood	0.316	0.0842	0.198	0.437**
	(-0.0959 - 0.727)	(-0.370 - 0.538)	(-0.260 - 0.657)	(0.0310 - 0.842)
Adult Respondent Characteristics				
Female (1 = yes)	-0.951***	0.843***		
	(-1.191 - -0.711)	(0.238 - 1.448)		
Urban (1 = yes)	2.800***	2.818***	2.974***	2.531***
	(2.480 - 3.119)	(2.449 - 3.188)	(2.676 - 3.272)	(2.170 - 2.893)
Adult height (cm)		0.0785***		
		(0.0681 - 0.0890)		
**Observations**	**104,359**	**33,998**	**56,137**	**48,222**
**R-squared**	**0.390**	**0.430**	**0.444**	**0.316**

*** $p < 0.01$, ** $p < 0.05$, * $p < 0.1$

Notes: The outcome variable in all regressions is attained education in years. All models are estimated using ordinary least squares, with 95 percent confidence intervals presented in parentheses. Standard errors are clustered at the country-region-survey year level. Column 1 presents results for the full sample, while columns 3 and 4 present results for the subsample of women and men, respectively.

Table A8: Multivariable Regressions for the Association between Av Cohort Average Under-5 HAZ and Adult Height, Excluding Rwanda

VARIABLES	(1)<br>Fully Adjusted<br>Pooled Sample	(2)<br>Fully Adjusted<br>WOMEN	(3)<br>Fully Adjusted<br>MEN
Under-5 HAZ	1.984***	1.953***	2.060***
	(1.023 - 2.944)	(0.925 - 2.981)	(0.595 - 3.524)
**Controls**			
Child Cohort Characteristics			
Pct. urban at childhood	-0.581	-0.625	-0.650
	(-1.956 - 0.793)	(-2.122 - 0.872)	(-2.828 - 1.528)
Pct. electricity at childhood	0.682*	0.522	1.028**
	(-0.0432 - 1.408)	(-0.560 - 1.604)	(0.0840 - 1.972)
Avg. mat. educ. at childhood	0.0269	0.0686	-0.377**
	(-0.291 - 0.345)	(-0.273 - 0.410)	(-0.660 - -0.0933)
Log income at childhood	-0.574**	-0.562**	-0.193
	(-1.019 - -0.129)	(-1.036 - -0.0866)	(-1.196 - 0.811)
Adult Respondent Characteristics			
Female (1 = yes)	-12.44***		
	(-12.93 - -11.95)		
Urban (1 = yes)	1.180***	1.207***	0.866**
	(0.959 - 1.401)	(0.984 - 1.430)	(0.143 - 1.589)
**Observations**	**32,459**	**29,576**	**2,883**
**R-squared**	**0.385**	**0.172**	**0.055**

*** $p < 0.01$, ** $p < 0.05$, * $p < 0.1$

Notes: The outcome variable in all regressions is adult height in cm. All models are estimated using ordinary least squares, with 95 percent confidence intervals presented in parentheses. Standard errors are clustered at the country-region-survey year level. Column 1 presents results for the full sample, while columns 2 and 3 present results for the subsample of women and men, respectively.

Table A9: Multivariable Regressions for the Association between Cohort Average Under-5 HAZ and Educational Attainment in Adulthood, Excluding Rwanda

VARIABLES	(1) Fully Adjusted Pooled Sample	(2) Fully Adjusted Pooled Sample + Adult Height	(3) Fully Adjusted Pooled Sample WOMEN	(4) Fully Adjusted Pooled Sample MEN
Under-5 HAZ	0.228	-0.378	0.258	0.284
	(-0.743 - 1.199)	(-1.818 - 1.062)	(-0.839 - 1.356)	(-0.594 - 1.162)
**Controls**				
Child Cohort Characteristics				
Pct. urban at childhood	-2.910***	-3.720***	-3.584***	-2.069***
	(-4.042 - -1.777)	(-5.443 - -1.998)	(-4.858 - -2.309)	(-3.156 - -0.982)
Pct. electricity at childhood	0.755	0.285	0.561	0.847*
	(-0.241 - 1.751)	(-0.921 - 1.491)	(-0.662 - 1.784)	(-0.0419 - 1.735)
Avg. mat. educ. at childhood	0.770***	1.173***	1.029***	0.464***
	(0.464 - 1.076)	(0.691 - 1.655)	(0.662 - 1.395)	(0.229 - 0.698)
Log income at childhood	0.279	0.0662	0.156	0.409*
	(-0.136 - 0.695)	(-0.426 - 0.558)	(-0.305 - 0.617)	(-0.00519 - 0.823)
Adult Respondent Characteristics				
Female (1 = yes)	-0.967***	0.945***		
	(-1.214 - -0.721)	(0.240 - 1.649)		
Urban (1 = yes)	2.813***	2.851***	2.988***	2.543***
	(2.489 - 3.137)	(2.472 - 3.230)	(2.686 - 3.291)	(2.176 - 2.911)
Adult height (cm)		0.0782***		
		(0.0678 - 0.0886)		
**Observations**	**101,330**	**32,459**	**54,500**	**46,830**
**R-squared**	**0.390**	**0.431**	**0.446**	**0.312**

*** $p < 0.01$, ** $p < 0.05$, * $p < 0.1$

Notes: The outcome variable in all regressions is attained education in years. All models are estimated using ordinary least squares, with 95 percent confidence intervals presented in parentheses. Standard errors are clustered at the country-region-survey year level. Column 1 presents results for the full sample, while columns 3 and 4 present results for the subsample of women and men, respectively.

Figure A1: Sample Construction Flowchart

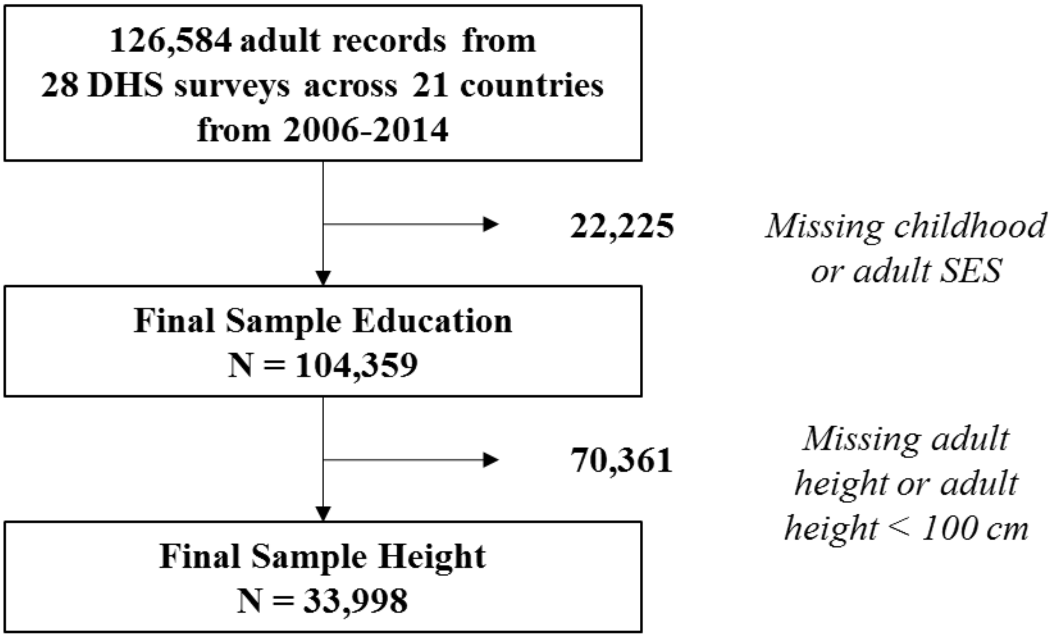

Figure A2: Map of Countries in the Final Sample

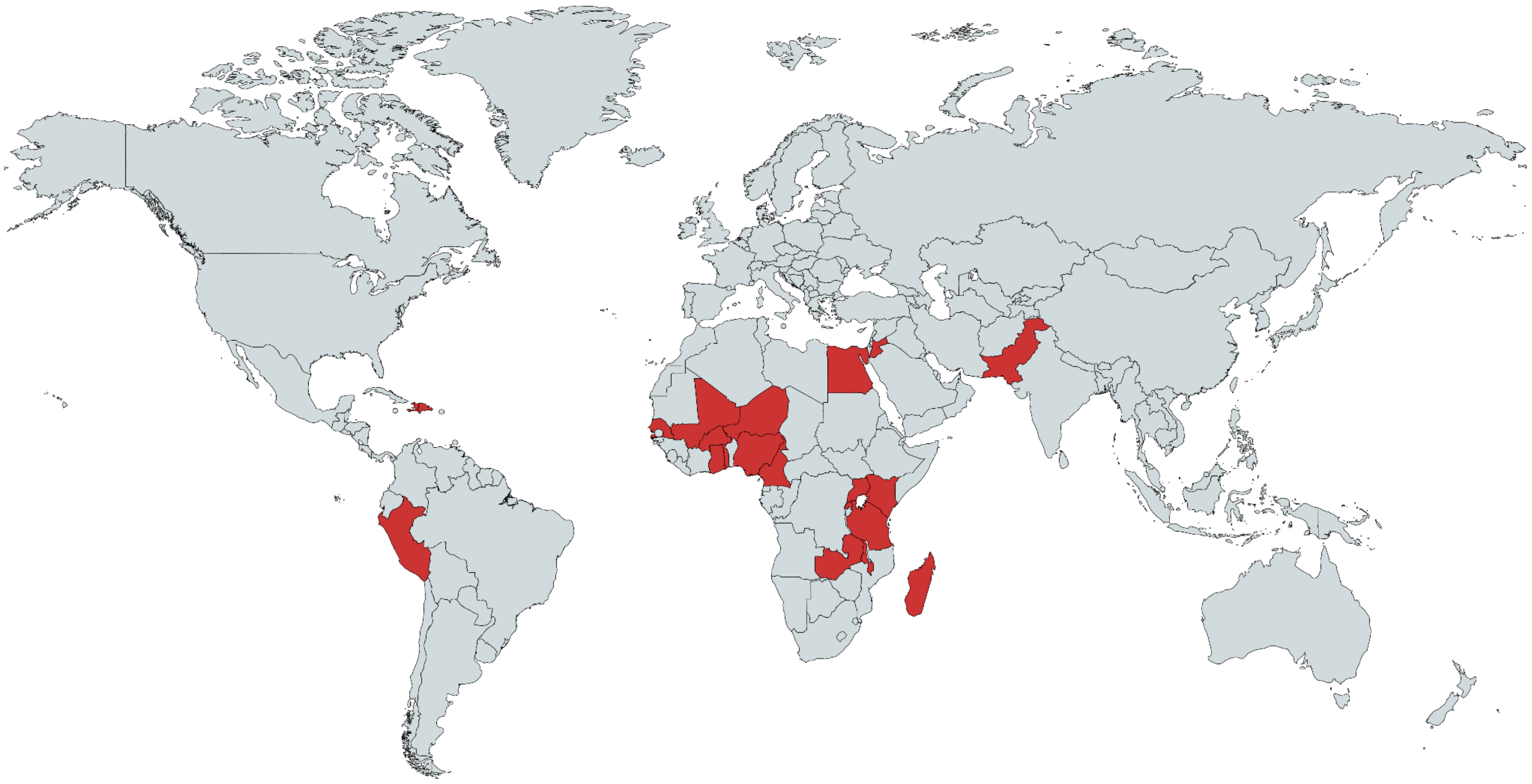

Figure A3: Cohort-Level Correlation between Childhood HAZ, Maternal Education, and Adult Female Educational Attainment: Kenya

A

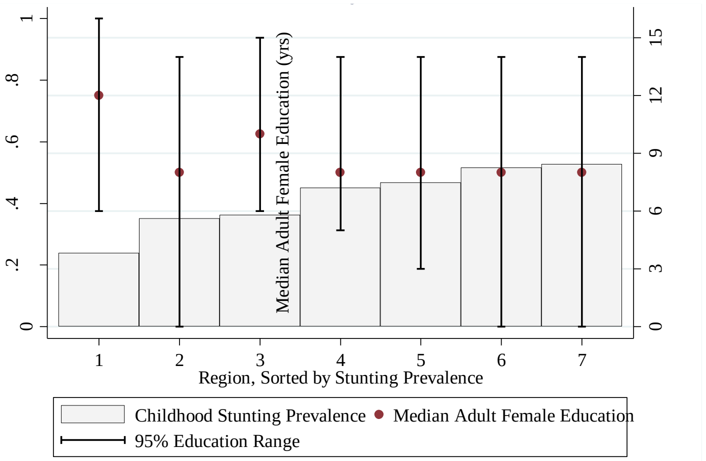

B

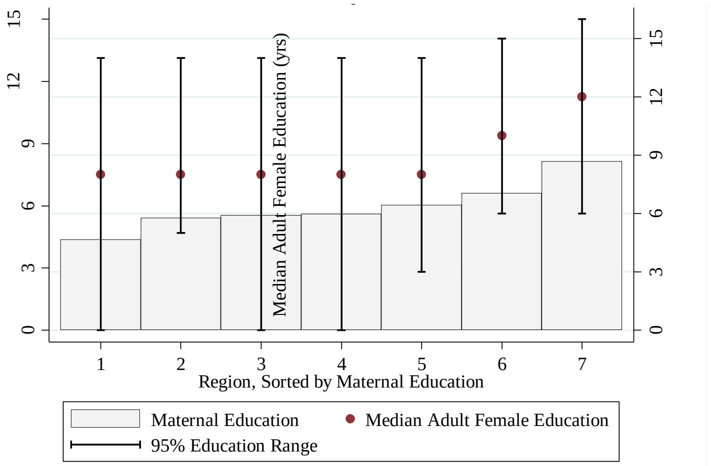

C

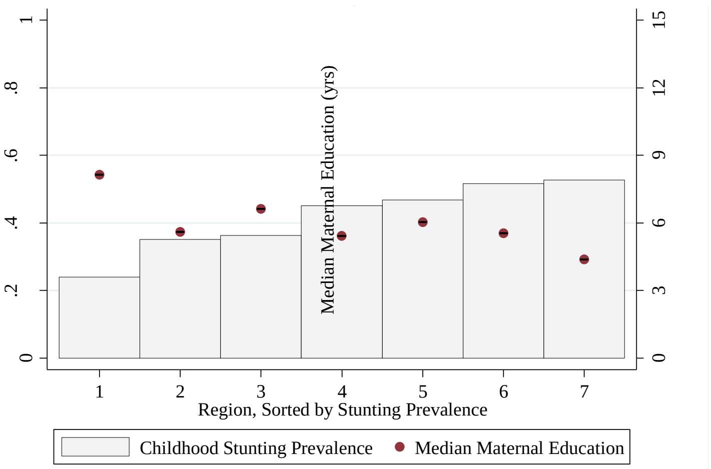

D

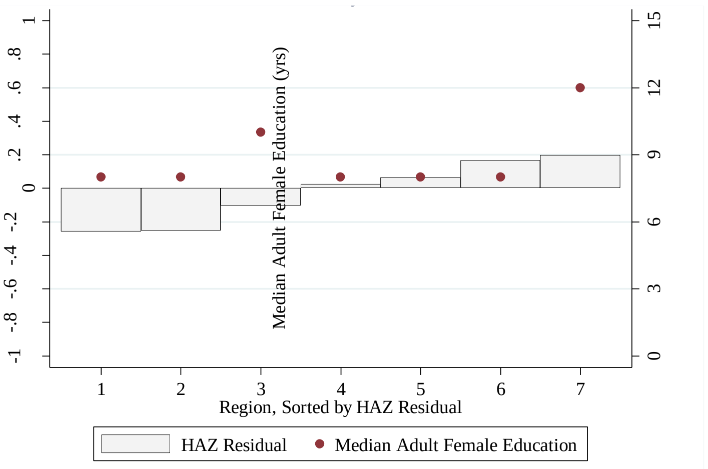

Supplement: Supplementary file 1 — Supplemental Materials. (PDF 827 kb) [file 12889_2019_7203_MOESM1_ESM.pdf]
